# 3D Printed Buccal Films for Prolonged-Release of Propranolol Hydrochloride: Development, Characterization and Bioavailability Prediction

**DOI:** 10.3390/pharmaceutics13122143

**Published:** 2021-12-13

**Authors:** Marija Jovanović, Miloš Petrović, Sandra Cvijić, Nataša Tomić, Dušica Stojanović, Svetlana Ibrić, Petar Uskoković

**Affiliations:** 1 Department of Materials Science and Engineering, Faculty of Technology and Metallurgy, University of Belgrade, Karnegijeva 4, 11120 Belgrade, Serbia; mpetrovic@tmf.bg.ac.rs (M.P.); duca@tmf.bg.ac.rs (D.S.); puskokovic@tmf.bg.ac.rs (P.U.); 2 Department of Pharmaceutical Technology and Cosmetology, Faculty of Pharmacy, University of Belgrade, Vojvode Stepe 450, 11221 Belgrade, Serbia; gsandra@pharmacy.bg.ac.rs (S.C.); svetlana.ibric@pharmacy.bg.ac.rs (S.I.); 3 Innovation Center, Faculty of Technology and Metallurgy, University of Belgrade, Karnegijeva 4, 111200 Belgrade, Serbia; ntomic@tmf.bg.ac.rs

**Keywords:** 3D printing, semi-solid extrusion, buccal films, gelatin, propranolol hydrochloride, prolonged-release, physiologically based simulations

## Abstract

Gelatin-polyvinylpyrrolidone (PVP) and gelatin-poly(vinyl alcohol) (PVA) mucoadhesive buccal films loaded with propranolol hydrochloride (PRH) were prepared by semi-solid extrusion 3D printing. The aim of this study was to evaluate the effects of the synthetic polymers PVP and PVA on thermal and mechanical properties and drug release profiles of gelatin-based films. The Fourier-transform infrared spectroscopy showed that hydrogen bonding between gelatin and PVP formed during printing. In the other blend, neither the esterification of PVA nor gelatin occurred. Differential scanning calorimetry revealed the presence of partial helical structures. In line with these results, the mechanical properties and drug release profiles were different for each blend. Formulation with gelatin-PVP and PRH showed higher tensile strength, hardness, and adhesive strength but slower drug release than formulation with gelatin-PVA and PRH. The in silico population simulations indicated increased drug bioavailability and decreased inter-individual variations in the resulting pharmacokinetic profiles compared to immediate-release tablets. Moreover, the simulation results suggested that reduced PRH daily dosing can be achieved with prolonged-release buccal films, which improves patient compliance.

## 1. Introduction

Three-dimensional (3D) printing, an additive manufacturing technology, has been widely used to prepare different formulations that provide a personalized approach in drug delivery and tissue engineering [1,2,3]. The main principle of this method is based on the fabrication of different forms from digital models via layer-by-layer addition of printed materials [4,5]. The advantage of 3D printing is the manufacturing of high-quality formulations with different drug release profiles. Different 3D printing methods have found application in the pharmaceutical field, such as fused deposition modeling (FDM), stereolithography (SLA), inkjet-based 3D printing, selective laser sintering (SLS), and semi-solid extrusion (SSE) [6]. Moreover, additive manufacturing is considered a particularly attractive method for developing oral dosage forms as well as mucosal drug delivery systems [7,8]. Herein, the properties of the active pharmaceutical ingredients (APIs) and polymers, the designed geometry/structure, and targeted drug release profiles influence the choice of a suitable method for 3D printing of the desired dosage form [4].

Semi-solid extrusion (SSE) 3D printing uses a paste as a material loading system. A syringe filled with a paste is pushed under pressure during the process, and the paste is deposited through a nozzle. Preparation of the paste is a simple procedure consisting of mixing the API with polymers and other excipients [4,6]. There is also no need for special post-processing, except drying or cooling. Because this method does not require high temperature or UV curing, thermosensitive and UV sensitive APIs can be used [6]. The processing parameters (temperature, flow rate of the paste, and size and speed of the print nozzle) and formulation parameters (viscosity, polymer concentration, fluidity, and solvent type) affect the properties of the final product [6].

The blending of polymers provides many possibilities for modifying materials’ characteristics and drug release profiles [9]. Natural or synthetic mucoadhesive polymers are usually used for buccal formulations because they increase the residence time of a dosage form on the application site and optimize drug delivery [7]. Gelatin is a natural polymer that is biocompatible, biodegradable, adhesive, non-toxic, and affordable. Because of these properties, gelatin is used in the pharmaceutical and biomedical fields for drug delivery and tissue engineering [10]. Gelatin is often blended with other polymers to obtain better material properties such as mechanical behavior and printability. Polyvinylpyrrolidone (PVP) and poly(vinyl alcohol) (PVA) are commonly employed polymers in pharmaceutical technology for manufacturing tablets, granules, hydrogels, nanofibers, as well as films and coatings [11,12]. They have a good film-forming ability and adhesiveness and can interact with hydrophilic materials like gelatin [7,11,12].

Drug delivery through buccal mucosa is an attractive route of drug administration because it enables the API to avoid the hepatic first-pass metabolism and degradation in the gastrointestinal (GI) tract [7,13]. Therefore, the bioavailability of some drugs can be increased by using the buccal dosage forms [13]. The oral mucosa has very good vascularization, and the API absorbed there directly enters systemic circulation [13,14]. This application route is convenient and accessible, and the buccal dosage form can be easily removed if needed [15]. In addition, patient compliance is improved, especially for the geriatric and pediatric population, as well as for patients with gastroesophageal disorders or patients having trouble swallowing [15]. In the process of developing buccal films, some important material properties have to be considered: biocompatibility, blending behavior, and mucoadhesion of the used polymers, as well as molecular weight, distribution coefficient (logD), lipophilicity (logP), solubility, and permeability of the API [7]. Buccal films can be developed as immediate-release (IR) or prolonged-release (PR) formulations. Buccal films can also be used for local diseases of the oral cavity [16] as part of a preventive and specific treatment strategy for gingivitis [17] and periodontitis [18] or to obtain systemic therapeutic effect [19,20,21]. These films should have the appropriate size and thickness to be used on the buccal mucosa [15]. Mechanical properties and mucoadhesive behavior should provide the flexibility, softness, elasticity, and resistance to breakage of films during manufacturing and application [15,22].

Propranolol hydrochloride (PRH), a model drug used in this study, is a non-selective β-adrenergic blocker. It has been used in the therapy of cardiovascular diseases such as hypertension [21], arrhythmias [23], and many others. It can also be used to treat anxiety [24], migraine [19], and tremor [25]. However, PRH is a drug that undergoes extensive first-pass metabolism that results in reduced bioavailability following peroral administration, so only approximately one-quarter of the dose reaches systemic circulation [19]. For this reason, the buccal route of administration is a suitable alternative for PRH formulations [26].

In this work, gelatin-based films for buccal delivery of PRH with different polymer blends were 3D printed using semi-solid extrusion to explore the effect of the synthetic polymers (PVP and PVA) on the films’ mechanical and mucoadhesive properties, drug release profile, and the corresponding buccal drug absorption. The prepared buccal films were evaluated using various physicochemical, mechanical, and biopharmaceutical in vitro and in silico characterization tools.

## 2. Materials and Methods

### 2.1. Materials

Type A gelatin from porcine skin (~300 g Bloom) (GA), polyvinylpyrrolidone (K30) (PVP), and poly(vinyl alcohol) (Mw 85,000–124,000) (PVA) were obtained commercially from Sigma-Aldrich (Sigma-Aldrich Co., St. Louis, MO, USA). Glycerol 85%, used as a plasticizer, was obtained from Zorka Pharma (Zorka Pharma HEMIJA d.o.o., Sabac, Serbia). Propranolol hydrochloride (PRH) (Galenika a.d., Belgrade, Serbia), which was used as a model substance, was Ph. Eur. 10 grade. Deionized water (DI) (resistance of 18 MW cm) was used to prepare the solutions. In vitro drug release and mucoadhesion studies were carried out in simulated saliva of the following composition: disodium hydrogen phosphate, potassium dihydrogen phosphate, and purified water (pH = 6.8 was adjusted with 0.1 M hydrochloric acid) [27]. The mucoadhesion studies used the mucin from a porcine stomach, Type II (Sigma-Aldrich Co., St. Louis, MO, USA).

### 2.2. Methods

#### 2.2.1. Preparation of 3D Printed Films

The buccal films were prepared by semi-solid extrusion 3D printing. First, a 10% *w/w* gelatin solution was made by dissolving gelatin and 10% glycerol (on a gelatin dry basis) in distilled water. The solution was then stirred for 2 h at 50 °C. For PRH loaded paste, this procedure was followed by adding 25% PRH (on a gelatin dry basis) and stirring for 3 h at 50 °C to get a homogenous solution. For gelatin solutions blended with PVP or PVA, 10% PVP or PVA solutions were made separately; polymer and 10% glycerol (on a dry polymer basis) were added to distilled water and were stirred for 2 h at 50 °C. When homogenous polymer solutions were obtained, 10% gelatin and PVP or PVA solutions were mixed at a ratio of 1:1 and stirred again for 3 h at 50 °C to get a good blend. The procedure for PRH loaded paste was the same; after obtaining polymer blend solutions, 25% PRH (on a dry polymer basis) was added and stirred for 3 h at 50 °C to get a homogenous solution. Finally, each formulation solution (Table 1) was transferred into a syringe and kept at room temperature overnight for gelatination.

Before 3D printing, the syringe filled with paste was kept at 36 °C to obtain good printing properties [1,3]. Ultimaker 2+ printer (Ultimaker B.V., Utrecht, The Netherlands) adapted with Discov3ry paste extruder (Structur3d Printing, Kitchener-Waterloo, ON, Canada) was used for 3D printing. The pastes were extruded through a 0.84 mm internal diameter nozzle tip, moving at a 15 mm/min speed at room temperature. The films were printed in the shape of 60 mm × 15 mm × 0.5 mm strips. After printing, they were left to dry at room temperature for a day. The dried films were stored in a desiccator (NaBr saline saturated solution, relative humidity = 58%) at room temperature until analysis [26].

#### 2.2.2. Fourier-Transform Infrared Spectroscopy (FTIR)

The formation of new chemical bonds between films’ components during processing was observed by Fourier-transfer infrared spectroscopy (FTIR) using a Nicolet 6700 spectrometer (Thermo Fisher Scientific, Waltham, MA, USA) in the attenuated total reflectance (ATR) mode. The equipment had a single bounce 45 °F Golden Gate ATR accessory with a diamond crystal and an electronically cooled DTGS detector. The spectra were recorded within 64 scans, with a resolution of 4 cm^−1^, and processed by OMNIC software in the wavenumber range from 4000 cm^−1^ to 500 cm^−1^.

#### 2.2.3. Differential Scanning Calorimetry (DSC)

Thermal properties of starting materials, polymer blends, and printed films loaded with PRH were analyzed with differential scanning calorimetry (DSC) (Q10, TA Instruments, Crawley, UK). Measurements were performed in the temperature range from 30 °C to 200 °C with a heating rate of 10 °C/min. Samples of 6–9 mg were investigated under nitrogen flow of 50 mL/min. The characteristic temperatures were determined in the accompanying software [26,28].

#### 2.2.4. Mechanical Properties

##### Tensile Test

The tensile test of films was performed on Texture Analyzer Shimadzu EZ Test LX (Shimadzu, Kyoto, Japan) equipped with a 500 N load cell, at room temperature, with a strain rate of 5 mm/min. The exact dimensions of the cross-section area and gauge length were accurately determined before each measurement. Tensile strength and modulus of elasticity were calculated by software Trapezium (Shimadzu, Kyoto, Japan). A minimum of three measurements was made for each film formulation, and average values were calculated.

##### Microindentation

The microindentation experiments were performed on Texture Analyzer Shimadzu EZ Test LX (Shimadzu, Kyoto, Japan) with a 500 N load cell and a spherical indenter with a d = 4 mm diameter. In each experiment, the load was set to continuously increase by 0.25 N/s until it reached 5 N. The maximum load was maintained for 20 s and then gradually decreased by 0.25 N/s until the full release. The values of force, time, and relative position of the indenter were measured. The microindentation was performed on a minimum of three positions on each film formulation to reduce the influence of possible inhomogeneity and to obtain more realistic results.

Microindentation is a promising method for evaluating the dynamic behavior of buccal and other drug-loaded thin films. From load (P) vs. depth (h) curves, it is possible to obtain values for hardness (H) and reduced elastic modulus (E_r_) according to the Oliver–Pharr method [29,30]:

(1) $$H=\frac{P_{\max}}{A}$$

(2) $$E_{r}=\frac{S}{2}\cdot \sqrt{\frac{\pi }{A}}$$

where P_max_ represents the maximum load and S is the stiffness, i.e., the slope of the unloading curve, which was found at maximum displacement/load (h_max_, P_max_):

(3) $$S=\frac{\mathrm{dP}}{\mathrm{dh}}.$$

Additionally, A represents the projected area of the indenter impression and, given the spherical shape of the indenter, it was calculated as:

(4) $$A=\left({\mathrm{dh}}_{c}-h_{c}^{2}\right)\cdot \pi$$

where h_c_ is the contact depth that can be determined as:

(5) $$h_{c}=h_{\max}-\frac{P_{\max}}{S}.$$

The corrective parameter (ε = 0.75) for the spherical shape of the indenter was also used in the analysis [29].

#### 2.2.5. Mucoadhesive Properties

Mucoadhesion tests were performed on Texture Analyzer Shimadzu EZ Test LX (Shimadzu Corporation, Kyoto, Japan) with a 500 N load cell, as in our previous research [26]. Obtained cylindrically cut films with a 10 mm diameter, fixed on a movable metal rod, were in contact with mucin disc with a 13 mm diameter, previously soaked in simulated saliva, for 60 s with the applied force of 1 N. The rod was then lifted with the constant velocity of 0.1 mm/s, and data for force, time, and stroke were recorded. The tests were stopped when the film got fully separated from the mucin disk. The area under the curve (AUC) was calculated from the force vs. stroke plot as the work of mucoadhesion [31]. Each experiment was carried out at least three times per film formulation.

#### 2.2.6. Field Emission Scanning Electron Microscope (FESEM)

The structure and morphology of obtained films were observed by FESEM TESCAN MIRA 3 (Tescan Orsay Holding, a.s., Brno-Kohoutovice, Czech Republic). Fractured surfaces of the films were sputtered with gold.

#### 2.2.7. Drug Content Uniformity

Three samples from different places of each film formulation were cut in 15 mm × 15 mm × 0.5 mm samples. The samples were then transferred into a 250 mL volumetric flask and dissolved in simulated saliva (pH = 6.8) [20]. After that, the solutions were filtered through a 0.45 µm membrane filter (Millipore, Bedford, MA, USA), and the absorbance of PRH was measured on UV-Vis spectrophotometer (LLG-uniSPEC 2 Spectrophotometer, Lab Logistics Group GmbH, Meckenheim, Germany) at 319 nm. To determine the drug content uniformity of films, we used the same method as in our previous work [26].

#### 2.2.8. In Vitro Drug Release

The dissolution studies were performed using an orbital shaker (Orbital shaker PSU-10i, Grant-bio, Grant Instruments Ltd., Cambridge, UK) at 50 rpm in a thermostatic incubator (MBIN50 Microbiological Incubator, Colo Lab Experts, Novo Mesto, Slovenia) at 37 °C [32]. The dissolution medium was 100 mL of simulated saliva [32,33]. The dimensions of the buccal films were 15 × 15 × 0.5 mm. The buccal film samples were fixed at the bottom of the beaker with double-sided tape, enabling drug release solely from one side of the films. Aliquots were withdrawn at predetermined time points, and the same amount of fresh prewarmed medium was added. The samples were filtered through a 0.45 µm membrane filter (Millipore, Bedford, MA, USA) and analyzed by a UV-Vis spectrophotometer (LLG-uniSPEC 2 Spectrophotometer, Lab Logistics Group GmbH, Meckenheim, Germany) at 319 nm. For every formulation, the experiment was carried out in triplicate.

#### 2.2.9. Drug Release Kinetics

The release kinetics of the PRH from the buccal films were analyzed using the DDSolver software (Microsoft Excel add-in program) developed by Zhang et al. [34]. The cumulative drug release data were fitted with different mathematical models, including zero-order, first-order, Higuchi, and Korsmeyer–Peppas models [35,36,37]. The highest values of the coefficient R^2^ and the model selection criterion (MSC) were utilized to determine the best fitting model [37].

#### 2.2.10. Physiologically Based Simulations

Commercially available software GastroPlus™ (v. 9.7.0009, Simulations Plus Inc., Lancaster, CA, USA) was used to simulate PRH absorption and disposition following intraoral administration of the tested buccal films. For comparison purposes, the simulations were also performed for PRH IR tablets. Our previous publication [26] describes basic data about the software performance and the applied model, along with the drug-specific input data used for model construction. In brief, the selected parameters describing PRH physicochemical and pharmacokinetic properties, in conjunction with the software default physiological parameters values for human adults in the fasted state, were used to model drug transport and absorption through the oral cavity mucosa and/or intestinal membrane, and to estimate the corresponding plasma concentration-time profiles. Here, the software-integrated Advanced Compartmental Absorption and Transit (ACAT) model of the GI tract was used to simulate the bioperformance of IR tablets, whereas the adjunct Oral Cavity Compartmental Absorption and Transit (OCCAT) model was used to estimate drug absorption from the tested buccal films. As explained in our previous publication [26], the only adjusted physiological parameter referring to the OCCAT model was saliva production rate (Table 2). The additional OCCAT model parameters, specific for the tested buccal film formulations, are noted in Table 2. In order to describe drug release rates from the tested formulations, experimentally obtained dissolution data, fitted by a single-Weibull function, were used as inputs in the model. A Weibull function was used to obtain smoother profiles and allow for more accurate extrapolation of the data. In addition, the model assumed that the contact surface area of the film with buccal mucosa is constant (software default) and that drug release happens in one direction (which complied with the in vitro dissolution test settings). The simulations were performed for a single PRH dose (as buccal film or IR tablet) and for repeated drug dosing to estimate the expected plasma concentration in the steady state.

In order to account for inter-individual variations in the physiological and drug pharmacokinetic parameters, the simulations were performed for a virtual group of 50 subjects, using the Population Simulations software module. The selected input parameters were varied randomly within the predefined range in each simulation. This range was defined for each parameter based on the initial (base) value (initial physiological parameters were software default values) and software default coefficient of variation (CV%), using a log-normal distribution. The default CVs% were 20% for transit times in different GI compartments, the volume of distribution, distribution rate constants, and hepatic first-pass extraction, 30% for hepatic blood flow, 40% for clearance, and 10% for all other variables.

#### 2.2.11. Statistical Analysis of the Experimental Results

The data obtained by evaluating mechanical and mucoadhesive properties were also analyzed using a two-way analysis of variance (ANOVA) at a confidence level of *p* < 0.05. Polymer type and drug presence were used as independent variables (factors), whereas tensile strength, modulus of elasticity, hardness, reduced modulus, maximum force, and work of adhesion from respective tests were used as dependent variables. The statistical analysis was performed in the OriginPro software (OriginLab Corporation, Northampton, MA, USA).

## 3. Results and Discussion

### 3.1. Fourier-Transform Infrared Spectroscopy (FTIR)

**Figure 1:**
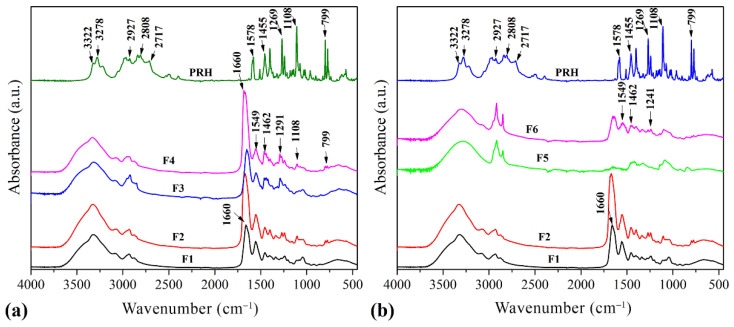
FTIR spectrum of 3D printed films (**a**) F1, F2, F3, F4, and PRH; (**b**) F1, F2, F5, F6, and PRH.

The FTIR spectrums of 3D printed films are presented in Figure 1. The FTIR spectrum of F1 showed intense bands at: 3320 cm^−1^ related to the OH stretching of internal hydrogen bonds overlapped with a band of N–H stretching, corresponding to Amide A; 1660 cm^−1^ which could be assigned to the vibration of the amide carbonyl group corresponding to Amide I; 1549 cm^−1^ associated with bending of the free amino groups coupled with CN stretching-Amide II; 1243 cm^−1^ related to NH bending-Amide III; 1045 cm^–1^ assigned to the characteristic C–O stretching band of glycerol [26,39,40].

The spectrum of the F3 blend is very similar to the spectrum of F1 with emphasized bands at 2929 and 2869 cm^−1^ due to the asymmetric and symmetric C–H stretching vibrations, respectively [39]. There is also another prominent peak at 1653 cm^−1^ due to C=O stretching vibration.

Due to the addition of PVP, the peak at 1460 cm^−1^ shifted to 1438 cm^−1^, which corresponds to in-plane bending vibration from C–H [41]. This shift could be attributed to hydrogen bonding between C=O in PVP and OH in gelatin [40]. A similar explanation could be given for the peak at 1243 cm^−1^, which shifted to 1290 cm^−1^ after established hydrogen bonding coupled with NH bending [40]. In the case of the F3 spectrum, these peaks are positioned at 1680 cm^−1^ and 1460 cm^−1^. Hence the peaks for functional groups of PRH and GA were unchanged as there was no interaction between the drug and gelatin [26]. Finally, the spectrum of F4 showed peaks at 1685, 1549, 1462, and 1291 cm^−1^ that revealed established hydrogen bonds between GA and PVP, whereas peaks from PRH (the C–O–C stretching in aryl alkyl ether a stretching band at 1108 cm^−1^, and the α-substituted naphthalene peak at 797 cm^−1^) were unchanged. Therefore, there was no interaction of the blend with PRH [26].

The spectrum of F5 showed peaks at 3299 cm^−1^ due to the stretching vibration of O–H bonds; at 2929 and 2869 cm^−1^ due to the asymmetric and symmetric C–H stretching vibrations, respectively; and a peak of medium intensity at 1462 cm^−1^ due to the bending vibrations of C–H bonds. [42,43]. Further, in the spectrum of F6, the observed peak at 1653 cm^−1^ is assigned to C=O stretching vibration. Finally, it can be seen that, in this spectrum, there are not any peaks at 1715–1730 cm^−1^, which are normally attributed to aromatic esters [44]. Therefore, this indicates that neither the esterification of PVA nor of the gelatin has taken place in formulation with PRH [43,44].

### 3.2. Differential Scanning Calorimetry (DSC)

**Figure 2:**
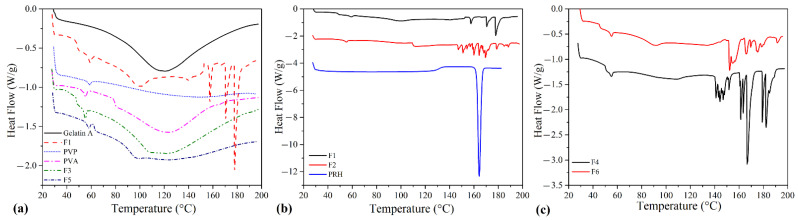
DSC thermograms of (**a**) Gelatin A, F1, PVP, PVA, F3, and F5; (**b**) F1, F2, and PRH; and (**c**) F4 and F6.

DSC analysis was performed to observe the thermal behavior of films as a consequence of intermolecular structure changes during processing. The DSC curves for starting materials Gelatin A, PVP, PVA, and films F1, F3 are presented in Figure 2a, whereas the curves for films F1, F2, and PRH are presented in Figure 2b. The curves for polymer blends loaded with PRH, F4 and F6, are presented in Figure 2c. Glycerol with a small hydrophilic molecule affects gelatin water absorption and also acts as a plasticizer. The molecules of glycerol are interspaced in a protein network and increase the distance between protein chains. In this work, glycerol was also added to establish better printability of films. In terms of thermal behavior, glycerol decreased the melting temperature (T_m_) of gelatin. It also made gelatin more sensitive to moisture, and an endothermic denaturation peak was observed on the thermogram of F1 [45,46,47,48]. Within the presented DSC curves, in the case of F1, two areas can be distinguished: the first one in the 45–60 °C range, in which glass transition temperature (T_g_) and triple helix denaturation of gelatin are observed, and the second one in the 160–180 °C range, in which peaks of cis and trans isomerism of glycine in gelatin are observed [26,48,49,50,51]. For formulations F3 and F5, it can be observed that the blending with PVP or PVA stabilizes gelatin, and then there is no cis-trans isomeric transformation.

Endothermic peaks in the range 140–185 °C are present in thermograms of drug-loaded formulations (F2, F4, and F6). The DSC thermogram of pure PRH shows a melting point at 163 °C [26]. However, the thermograms of drug-loaded formulations did not show the melting peak of the PRH, indicating that the 3D semi-solid extrusion method induced the amorphization of propranolol [27,52,53]. The addition of glycerol as a plasticizer leads to the depression of characteristic temperatures of polymers and the melting temperature of PRH, causing further decomposition from 146 °C for F2, 140 °C for F4, and 151 °C for F6. As is noted in our previous research [26], the drug’s amorphous form should be advantageous in terms of solubility and bioavailability.

### 3.3. Mechanical Properties

#### 3.3.1. Tensile Test

**Figure 3:**
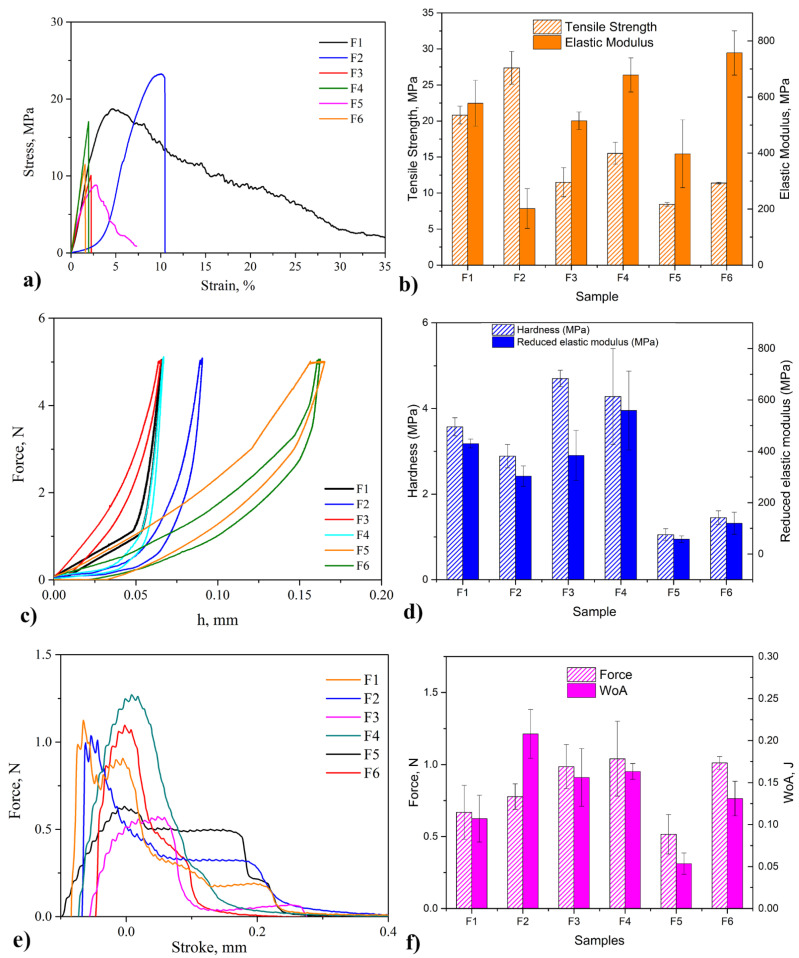
Results of: (**a**,**b**) tensile test; (**c**,**d**) microindentation; (**e**,**f**) mucoadhesion.

Results of tensile tests of all formulations of 3D printed films are given in a form of histograms (mean values) and as a diagram (for one representative film) in Figure 3a,b, respectively. In addition, the results of the ANOVA analysis are shown in Table 3. It can be seen that F1 showed quite satisfactory tensile strength, modulus of elasticity, toughness (area under the tensile curve), and breaking elongation, in line with previous research [26]. With the addition of the drug or when blended with PVP or PVA, toughness and breaking elongation decrease. Obtained results for blended films F3 and F5 are in agreement with literature data [39,44,54].

The tensile tests revealed that F6 showed 30% lower tensile strength and 11% higher modulus of elasticity than F4. That could be explained by the formation of hydrogen bonds between GA and PVP, which leads to higher tensile strength and decreased energy absorption ability during deformation [55]. Additionally, the presence and higher content of triple-helical structures improve mechanical properties. The influence of both polymer and the presence of drug on tensile strength and their interaction on elastic modulus were shown to be statistically significant (*p* < 0.05), as shown in Table 3.

#### 3.3.2. Microindentation

Microindentation is a suitable method for investigating visco-elastic properties of biopolymers, like gelatin [56,57,58,59]. The load-depth curves and obtained values for reduced modulus, E_r_, and hardness, H are presented in Figure 3c,d, respectively. The reduced modulus of F4 is approximately four times higher than that of F6, whereas the hardness is approximately three times higher. A different structure of these films, the higher content of triple-helical structure, and cis-trans isomeric transformation in F4 could explain this, as is supported by the DSC analysis. Gelatin is a product of collagen degradation. The higher content of disordered, coiled, and shortened collagen chains leads to greater probabilities of chain tangling and formation of helical structure bonding. With the higher bonding, a greater force is necessary to deform the gelatin-based film, and the stiffness increases as well [60]. The influence of polymer on hardness and reduced modulus were shown to be statistically significant (*p* < 0.05), as shown in Table 3.

### 3.4. Mucoadhesive Properties

Mucoadhesion is a significant property of buccal films; it describes how close a film is attached to the buccal mucus layer. The results of this research were analyzed from the fracture theory perspective, but the adhesive behavior of different formulations could be explained by other theories, such as electronic or charge, diffusion, wetting, and adsorption theories [61,62]. Force-stroke curves and results of tests—maximum detachment force and work of adhesion—are shown in Figure 3e,f, respectively.

It can be seen that the highest value of the adhesion force was obtained for F4, which agrees with other good mechanical properties of this formulation. Better attachment of F4 on the mucin can be explained by the formation of hydrogen bonds, the higher content of triple-helical structures, and the cis-trans isomeric transition during processing. Additionally, the electrostatic interaction between positively charged gelatin and negatively charged mucin could be modified by adding PRH and PVP or PVP. These molecules, according to their polarity, enhance or suppress this interaction [15,63]. F5 showed a lower adhesion force than F1, whereas F2 and F3 showed a higher one. Finally, F4 achieved a higher force than F6 because of the similar polarity of PVA and PRH. Work of adhesion correlates with the crosslinking of polymers and the possibility of liquid permeation in a polymer network. Among drug-loaded formulations, F2 has the highest value of the work, which implies the low density of crosslink [26]. The influence of polymer on the work of adhesion was shown to be statistically significant (*p* < 0.05), as shown in Table 3.

### 3.5. Field Emission Scanning Electron Microscope (FESEM)

**Figure 4:**
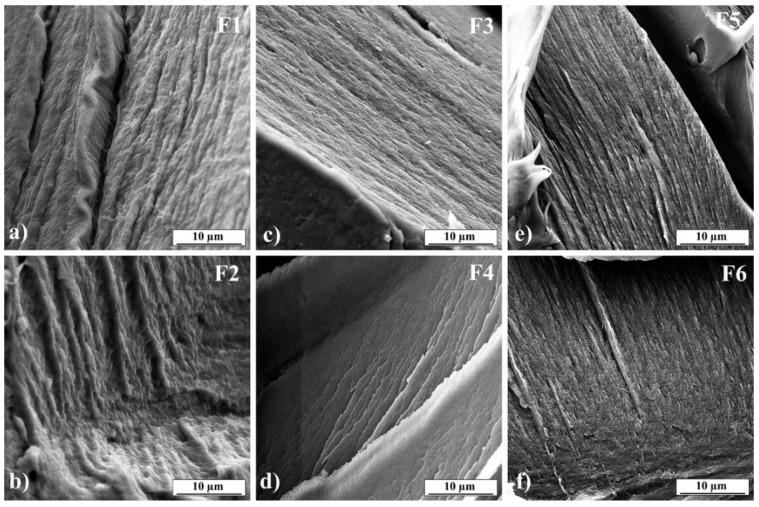
SEM images of 3D printed films: (**a**) F1, (**b**) F2, (**c**) F3, (**d**) F4, (**e**) F5, (**f**) F6.

SEM was used to examine the morphology of obtained films. SEM images are presented in Figure 4. It can be seen that all films have consistent structure without pores and show the surface’s roughness. However, sharper creases can be observed in the images of formulations F4 and F6 (Figure 4d,f, respectively). The creases could be explained by the different stiffness of triple-helix gelatin molecules compared to PVP or PVA molecules [64,65]. The presence of the undissolved drug was not observed, as can be seen in Figure 4b,d,f [66].

### 3.6. Drug Content Uniformity

The results of drug content uniformity are given in Table 4. Because they are within the limit of drug content uniformity, i.e., 85–115% [67], the buccal films have a uniform drug distribution.

### 3.7. In Vitro Drug Release

**Figure 5:**
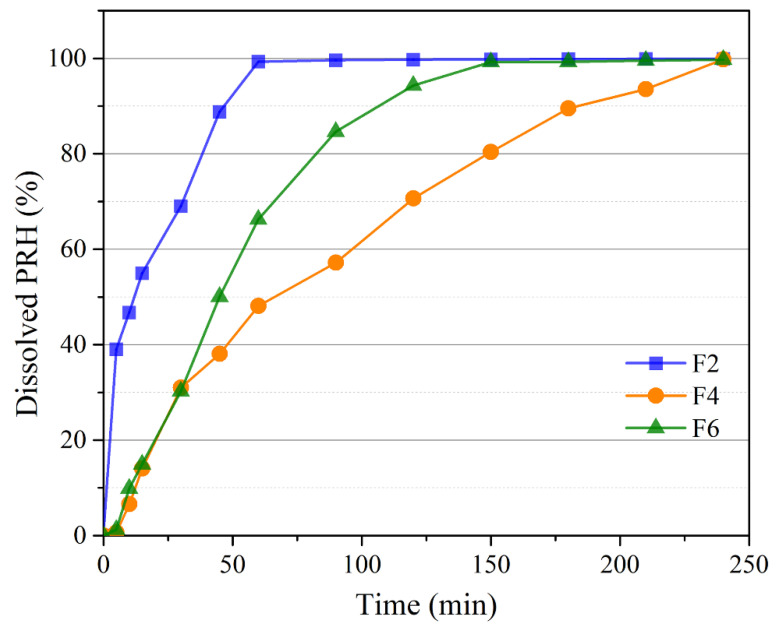
In vitro drug release.

Cumulative drug release profiles from all tested formulations are illustrated in Figure 5. During the test, all samples stayed fixed at the bottom of the beaker, and the sink conditions were maintained. It can be seen that complete PRH release from each formulation was achieved within 4 h. However, there were notable differences between the PRH release rates of different formulations. Formulation F2 demonstrated the fastest drug release (complete dissolution in 1 h), with initial burst PRH release (more than 50% in 15 min) followed by a somewhat slower dissolution rate. The other two formulations exhibited prolonged drug release, with complete PRH dissolution in 2.5 and 4 h for formulations F6 and F4, respectively.

In formulation F4, besides the formation of hydrogen bonds between molecules of the same polymer, there are also established hydrogen bonds between two different macromolecules (hydroxyl groups of the GA and carbonyl groups of PVP; amine groups of the GA and carbonyl groups of PVP). These hydrogen bonds cause the high cross-linked structure of the F4 formulation [40,64,68].

Due to the different polarity of molecules, this structure associated with PVP and PRH attraction slows down the liquid penetration in a polymer network and hence cause slower PRH release than in F2 and F6. As for formulation F6, a lower drug release rate when compared to formulation F2 can be explained by densely arranged molecules of gelatin and synthetic polymer PVA in this formulation [65]. On the contrary, in formulation F6, there are no new chemical bonds, and because molecules of PVA and PRH have similar polarity, this results in faster PRH release from F6 than from F4. The blending of GA with PVP or PVA makes it possible to prolong the PRH release time [44,69].

### 3.8. Drug Release Kinetics

The fitting results of the drug release profiles from 3D printed buccal films are shown in Table 5. The release model with the best fit is one with the highest values of R^2^ and MSC (a value greater than 2 indicates a good fit [34]). In this study, the best mathematical model for all formulations was first-order kinetic, which means that the release rate shows dependence on PRH concentration, and it is proportional to the drug remaining in the matrix [36,70]. The Korsmeyer–Peppas model also resulted in a good fit, which is why we calculated the release exponent (*n* value) to understand the PRH release mechanism from buccal films better [37]. Formulation F2 has an *n* value lower than 0.5, suggesting that the PRH release followed the Fickian diffusion mechanism. Formulations F4 and F6 have an *n* value between 0.5 and 1, meaning that the PRH release mechanism is both swelling and diffusion and follows anomalous or non-Fickian transfer [37,38,71].

### 3.9. Physiologically Based Simulations

**Figure 6:**
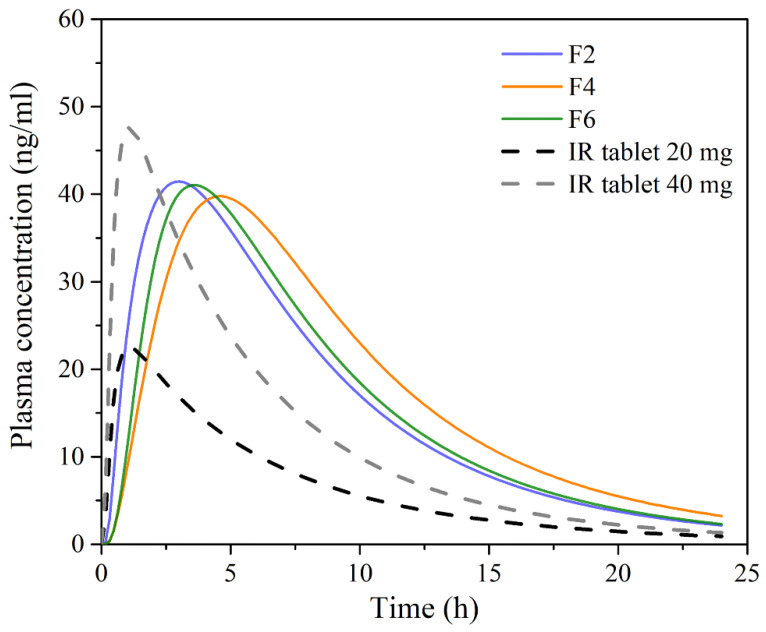
Mean predicted plasma concentration-time profiles following single dosing of the tested 20 mg PRH buccal films, and 20 mg and 40 mg IR tablets in 50 virtual subjects.

The simulation results for single-dosed 20 mg PRH buccal films, along with the data for PRH IR tablets containing different drug doses, are shown in Figure 6 and Table 6. The data in Figure 6 refer to the mean profiles for a virtual population of 50 subjects.

**Figure 7:**
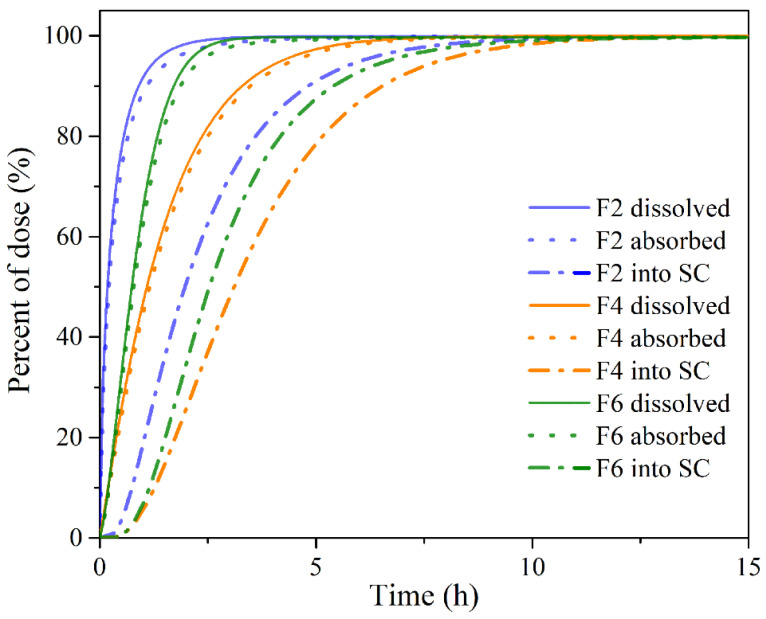
Predicted cumulative percent of PRH dissolved, absorbed, and entered into system circulation (SC) following single-dose administration of the tested buccal films.

The obtained results demonstrate certain differences in PRH absorption profiles following administration of the tested buccal films, mostly in terms of *t_max_*, whereas the differences in C_max_ and AUC values were not pronounced. The observed differences in the rate of drug absorption are caused by different PRH release rates from the tested formulations (Figure 5), i.e., slower drug release rate resulted in slower drug absorption. Following dissolution, the drug goes directly into the buccal epithelium through the contact surface area, resulting in a concomitant increase in the percent of drug absorbed into the buccal tissue (Figure 7). After the drug enters the buccal epithelium, it diffuses across the epithelium and lamina propria and eventually passes into the systemic circulation. The rate of these processes depends on the physiological characteristics of the buccal tissue and drug properties, such as diffusivity through the oral mucosa and F_ut_ value [72]. Therefore, the resulting cumulative percent of PRH that entered systemic circulation lags behind the percent of drug absorbed (Figure 7). However, drug uptake into the systemic circulation following administration of the tested buccal films is complete, in contrast to the limited drug bioavailability from IR tablets (due to the first-pass metabolism in the liver) (Table 6). Moreover, simulation results indicated that the extent of drug absorption (expressed as AUC) from the tested formulations lies in the range predicted for IR tablets containing a twice higher drug dose (Table 6). These outcomes imply that the same therapeutic effect may be achieved with reduced PRH doses in buccal films while causing fewer side effects.

**Figure 8:**
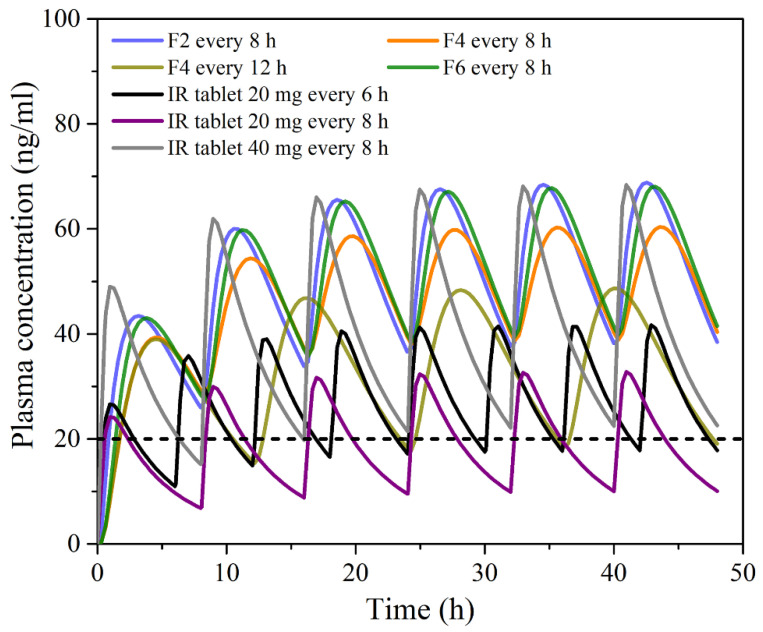
Mean predicted plasma concentration-time profiles following multiple dosing of the tested 20 mg PRH buccal films, and 20 mg and 40 mg IR tablets in 50 virtual subjects; the dotted line represents the minimum effective concentration.

In addition, simulations with repeated PRH dosing under different dosing regimens (Figure 8) revealed that buccal administration of the tested films might enable reduced PRH daily dosing compared to IR tablets, thus improving patient compliance. PRH therapeutic dose and dosing regimen depend on the indication, but the drug is most commonly given in divided doses 2–4 times per day [73,74]. For example, the recommended or the commercially available dose of PRH tablets for hypertrophic subaortic stenosis (thickened heart muscle) for adults is 20 to 40 mg three or four times per day [75]; for arrhythmias, hypertrophic obstructive cardiomyopathy and anxiety tachycardia, the dose for adults is in the range of 10 to 40 mg three or four times per day [76]. However, according to the simulations, even 3–4 times daily administration of 20 mg PRH IR tablets may not be able to maintain drug plasma concentration above the minimum effective concentration (MEC) of 20 ng/mL (preferably more) required to achieve inhibition of beta-adrenergic receptors [73,77]. Figure 8 shows only the mean data for a virtual population, meaning that the risk of subtherapeutic dosing is expected to be even higher when inter-individual differences in PRH pharmacokinetics are considered. Opposite to IR tablets, the investigated buccal films dosed three times per day are expected to maintain PRH plasma concentration in a “safe space” above the MEC (Figure 8). Additionally, it was observed that formulation F4, dosed two times per day, should provide a greater extent of PRH absorption in comparison to an IR tablet containing the same drug dose but administered more frequently, i.e., four times per day.

Another observation regarding PRH absorption from the tested buccal films is that the predicted pharmacokinetic parameters (i.e., C_max_, AUC_0–∞_ and drug bioavailability) for the virtual population are less prone to variations between subjects compared to the values predicted for IR tablets (Table 6). Namely, the simulated data suggest that inter-individual variability in C_max_ and AUC would be decreased by cc. 40% when switching from PRH IR tablets to the tested buccal film formulations.

## 4. Conclusions

Three-dimensional printing, a modern technique for advanced pharmaceutical formulations, was used to prepare buccal mucoadhesive gelatin-based films. In order to improve the mechanical properties of these films loaded with PRH and increase drug bioavailability, two blends of gelatin (with PVP and PVA) were prepared as the polymer matrix. The influence of polymer blend type on the films’ printability, structure, thermal and mechanical properties of the films, and drug release profile was investigated. Critical properties of 3D printed films, such as tensile strength, modulus of elasticity, hardness, mucoadhesion, drug content, and drug release rate, have been discussed, as these properties are significant determinants of the mucoadhesive buccal films’ behavior during application and the drug bioavailability from these films.

The presence of PVP in the blend and consequently in formulation F4 improves thermal and mechanical properties, exhibits a good mucoadhesion, and enables the prolonged release of PRH with the lowest rate. A highly cross-linked structure caused by the established hydrogen bonds between two different macromolecules (gelatin and PVP) could explain this behavior. On the contrary, in the case of the F6 formulation, no additional chemical bonding between gelatin and PVA occurred. Therefore, the drug release rate in this formulation is higher than in F4 but lower than in F2. This difference could be ascribed to densely arranged molecules of gelatin and synthetic polymer PVA. The addition of PVA to the gelatin-based matrix leads to thermal stability, a higher modulus of elasticity, and better mucoadhesion.

The simulation results revealed that PRH bioavailability, following application of the tested buccal films, is expected to be approximately three times higher than from IR tablets because the drug absorbed from the oral cavity bypasses first-pass extraction in the liver. In addition, the simulations suggested reduced dosing frequency of buccal films with prolonged PRH release (F4 and F6) in comparison to conventional IR tablets, which may increase patient compliance. As another advantage, population simulations indicated decreased inter-individual variations in PRH pharmacokinetics following administration of the buccal films.

By blending gelatin with PVP or PVA, we obtained PRH loaded buccal mucoadhesive films with good mechanical and mucoadhesive properties and prolonged drug release. The presented results indicate that with proper choice of synthetic polymer and blend composition, it is possible to tune mechanical properties as well as drug release profiles, depending on the application requirements. Finally, good printability was achieved, and the semi-solid 3D printing method was successfully introduced in buccal film preparation.

## Figures and Tables

**Table 1:** Composition of paste formulation for mucoadhesive buccal films.

Formulations	F1	F2	F3	F4	F5	F6
Ingredients						
Gelatin, type A	6 g	6 g	3 g	3 g	3 g	3 g
PVP			3 g	3 g		
PVA					3 g	3 g
Glycerol 85%	0.6 g	0.6 g	0.6 g	0.6 g	0.6 g	0.6 g
PRH		1.5 g		1.5 g		1.5 g
Purified water	ad 60 g	ad 60 g	ad 60 g	ad 60 g	ad 60 g	ad 60 g

**Table 2:** The study-specific input parameters in the OCCAT model.

OCCAT Parameter	Value
Formulation type	Buccal patch ^a^
Drug dose	20 mg ^a^
Film surface area	2.25 cm^2 a^
Oral transit model	Normal swallowing ^a^
Saliva production rate (base value)	0.6 mL/min ^b^
Fraction unbound in oral tissue (F_ut_)	0.21 ^c^
Drug diffusivity through oral mucosa	7.21 × 10^−7^ cm^2^/s ^c^

^a^ applies for the investigated buccal films; ^b^ optimized value [38]; ^c^ calculated using the software integrated equations.

**Table 3:** The results of ANOVA tests for the obtained mechanical and mucoadhesive properties.

	**Tensile Strength**		**Hardness**		**Strength of Adhesion**	
**Factor**	***F* Value**	***p* Value**	***F* Value**	***p* Value**	***F* Value**	***p* Value**
Polymer	42.94	<0.001	21.93	<0.001	1.96	0.173
Drug	11.23	0.007	0.34	0.570	2.91	0.107
Interaction	0.69	0.525	0.66	0.535	1.12	0.350
	**Elastic Modulus**		**Reduced Modulus**		**Work of Adhesion**	
**Factor**	***F* Value**	***p* Value**	***F* Value**	***p* Value**	***F* Value**	***p* Value**
Polymer	3.92	0.055	12.71	0.001	4.27	0.032
Drug	0.50	0.494	0.33	0.575	8.56	0.010
Interaction	10.79	0.003	1.89	0.194	1.78	0.201

*F* value represents the ratio of between-groups variance and within-groups variance; *p*-value determines whether the observed test results are statistically significant.

**Table 4:** Drug content uniformity.

Formulations	F2	F4	F5
Drug content percentage	98.27 ± 2.39	97.58 ± 3.65	95.74 ± 3.17

**Table 5:** Drug release kinetics.

Model	Zero Order		First Order		Higuchi Model		Korsmeyer–Peppas Model		
Criteria	R^2^	MSC	R^2^	MSC	R^2^	MSC	R^2^	MSC	*n* Value
Formulation									
**F2**	0.6777	1.2998	0.9682	2.6653	0.5167	0.0553	0.9259	2.1537	0.215
**F4**	0.8940	1.9315	0.9902	4.3081	0.9655	3.0539	0.9858	3.7843	0.630
**F6**	0.7281	0.9723	0.9797	3.5694	0.9256	2.2687	0.9268	2.7314	0.528

R^2^—coefficient of determination; MSC—model selection criterion; *n* value—release exponent, indicating the drug-release mechanism.

**Table 6:** Predicted pharmacokinetic parameters following single-dose administration of the tested 20 mg PRH buccal films and 20 mg and 40 mg IR tablets in 50 virtual subjects.

Formulation (Dose)	C_max_ (ng/mL)		*t_max_* (h)		AUC_0–∞_ (ng h/mL)		F (%)	
	Mean (Range)	CV%	Mean (Range)	CV%	Mean (Range)	CV%	Mean (Range)	CV%
F2 (20 mg)	42.13 (23.16–71.36)	26.59	3.10 (1.86–4.58)	19.43	404.87 (197.69–803.03)	34.80	99.89 (99.88–99.90)	2.62 × 10^−3^
F4 (20 mg)	40.72 (21.43–68.54)	24.26	4.78 (3.24–7.08)	16.98	451.74 (210.46–808.55)	31.49	99.99 (99.93–100.00)	1.08 × 10^−2^
F6 (20 mg)	41.67 (23.08–70.61)	26.56	3.70 (2.56–5.16)	15.32	404.10 (197.32–801.51)	34.80	99.70 (99.69–99.71)	2.77 × 10^−3^
IR tablet (20 mg)	22.81 (0.74–54.38)	45.42	1.12 (0.65–1.62)	19.35	168.02 (4.44–461.53)	53.45	35.24 (1.00–54.92)	33.82
IR tablet (40 mg)	48.14 (1.23–100.97)	44.76	1.08 (0.74–1.62)	16.99	319.63 (5.36–1088.80)	57.97	33.12 (1.32–57.57)	36.38

C_max_—maximum plasma concentration; *t_max_*—time to reach C_max_; AUC—area under the plasma concentration-time curve; F—bioavailability, CV%—coefficient of variation.

## Data Availability

Data are contained within the article.
